# Characteristics of a Multistate Outbreak of Lung Injury Associated with E-cigarette Use, or Vaping — United States, 2019

**DOI:** 10.15585/mmwr.mm6839e1

**Published:** 2019-10-04

**Authors:** Cria G. Perrine, Cassandra M. Pickens, Tegan K. Boehmer, Brian A. King, Christopher M. Jones, Carla L. DeSisto, Lindsey M. Duca, Akaki Lekiachvili, Brandon Kenemer, Mays Shamout, Michael G. Landen, Ruth Lynfield, Isaac Ghinai, Amy Heinzerling, Nathaniel Lewis, Ian W. Pray, Lauren J. Tanz, Anita Patel, Peter A. Briss, Lung Injury Response Epidemiology/Surveillance Group, Lung Injury Response Epidemiology/Surveillance Group, Jennifer Adjemian, Minal Amin, Jose Aponte, Vaughn Barry, Diane Browning, Jordan Cates, Gyan Chandra, Karen Chang, Katelyn Chiang, Jennifer Chevinsky, Augustina Delaney, Angela Dunn, Molly Evans, Victoria Fields, Aaron Fleischauer, Macarena Garcia, Caitlin Green, Arianna Hanchey, Kathleen Hartnett, Brooke Hoots, Asad Islam, Charlotte Kaboré, Vikram Krishnasamy, Mohammed Lamtahri, Jennifer Layden, Dana Meany-Delman, Jonathan Meiman, Christina Mikosz, Maureen Miller, Yousra Mohamoud, Erin Moritz, Varsha Neelam, David Nitschke, Kevin O’Laughlin, Samantha Olson, Tia Rogers, Nicki Roth, Phil Salvatore, Alana Vivolo-Kantor, Angela Werner, Jason Wilken

**Affiliations:** ^1^National Center for Chronic Disease Prevention and Health Promotion, CDC; ^2^National Center for Injury Prevention and Control, CDC; ^3^National Center for Environmental Health, CDC; ^4^Epidemic Intelligence Service, CDC; ^5^New Mexico Department of Health; ^6^Minnesota Department of Health; ^7^Illinois Department of Public Health; ^8^California Department of Public Health; ^9^Utah Department of Health; ^10^Wisconsin Department of Health Services; ^11^North Carolina Department of Health and Human Services; ^12^National Center for Immunization and Respiratory Diseases, CDC.; Center for Surveillance, Epidemiology, and Laboratory Services, CDC; National Center for Immunization and Respiratory Diseases, CDC; Center for Surveillance, Epidemiology, and Laboratory Services, CDC; Epidemic Intelligence Service, National Center for Injury Prevention and Control, CDC; Northrop Grumman; Epidemic Intelligence Service, National Center for Immunization and Respiratory Diseases, CDC; National Center for Chronic Disease Prevention and Health Promotion, CDC; Epidemic Intelligence Service, National Center for Chronic Disease Prevention and Health Promotion, CDC; National Center for Chronic Disease Prevention and Health Promotion, CDC; Epidemic Intelligence Service, National Center for Chronic Disease Prevention and Health Promotion, CDC; G2S Corporation; Utah Department of Health; National Center for Injury Prevention and Control, CDC; Epidemic Intelligence Service, National Center on Birth Defects and Developmental Disabilities; CDC; North Carolina Department of Health and Human Services; Center for Preparedness and Response, CDC; Center for Surveillance, Epidemiology, and Laboratory Services, CDC; National Center on Birth Defects and Developmental Disabilities, CDC; National Center for Environmental Health, CDC; Center for Surveillance, Epidemiology, and Laboratory Services, CDC; National Center for Injury Prevention and Control, CDC; Center for Surveillance, Epidemiology, and Laboratory Services, CDC; National Center for Chronic Disease Prevention and Health Promotion, CDC; National Center for Injury Prevention and Control, CDC; Center for Surveillance, Epidemiology, and Laboratory Services, CDC; Illinois Department of Public Health; National Center on Birth Defects and Developmental Disabilities; Wisconsin Department of Health Services; National Center for Injury Prevention and Control, CDC; Epidemic Intelligence Service, National Center for Chronic Disease Prevention and Health Promotion, CDC; National Center for Chronic Disease Prevention and Health Promotion, CDC; National Center for Environmental Health, CDC; National Center on Birth Defects and Developmental Disabilities, CDC; Center for Surveillance, Epidemiology, and Laboratory Services, CDC; Epidemic Intelligence Service, National Center for Emerging and Zoonotic Infectious Diseases, CDC; , G2S Corporation; Epidemic Intelligence Service, National Center for Injury Prevention and Control, CDC; Eagle Medical Services; Epidemic Intelligence Service, National Center for Injury Prevention and Control, CDC; National Center for Injury Prevention and Control, CDC; National Center for Environmental Health, CDC; California Department of Public Health, Center for Preparedness and Response, CDC; Council of State and Territorial Epidemiologist Vaping-Associated Pulmonary Injury Task Force.

Electronic cigarettes (e-cigarettes), also called vapes, e-hookas, vape pens, tank systems, mods, and electronic nicotine delivery systems (ENDS), are electronic devices that produce an aerosol by heating a liquid typically containing nicotine, flavorings, and other additives; users inhale this aerosol into their lungs (*1*). E-cigarettes also can be used to deliver tetrahydrocannabinol (THC), the principal psychoactive component of cannabis (*1*). Use of e-cigarettes is commonly called vaping. Lung injury associated with e-cigarette use, or vaping, has recently been reported in most states (*2*–*4*). CDC, the Food and Drug Administration (FDA), state and local health departments, and others are investigating this outbreak. This report provides data on patterns of the outbreak and characteristics of patients, including sex, age, and selected substances used in e-cigarette, or vaping, products reported to CDC as part of this ongoing multistate investigation. As of September 24, 2019, 46 state health departments and one territorial health department had reported 805 patients with cases of lung injury associated with use of e-cigarette, or vaping, products to CDC. Sixty-nine percent of patients were males, and the median age was 23 years (range = 13–72 years). To date, 12 deaths have been confirmed in 10 states. Among 514 patients with information on substances used in e-cigarettes, or vaping products, in the 30 days preceding symptom onset, 76.9% reported using THC-containing products, and 56.8% reported using nicotine-containing products; 36.0% reported exclusive use of THC-containing products, and 16.0% reported exclusive use of nicotine-containing products. The specific chemical exposure(s) causing the outbreak is currently unknown. While this investigation is ongoing, CDC recommends that persons consider refraining from using e-cigarette, or vaping, products, particularly those containing THC. CDC will continue to work in collaboration with FDA and state and local partners to investigate cases and advise and alert the public on the investigation as additional information becomes available.

State health departments, the Council of State and Territorial Epidemiologists (CSTE), and CDC have developed definitions for confirmed and probable cases* and medical chart abstraction and case interview forms. The case definition, forms, and instructions for reporting cases were disseminated to all state health departments in late August 2019. Patients with cases of lung injury associated with e-cigarette use, or vaping, had 1) a history of e-cigarette use, vaping, or dabbing (vaping concentrated marijuana) within 90 days before symptom onset; 2) imaging studies showing lung injury; 3) absence of evidence of infection (confirmed cases) or infection not thought to be the sole cause of the lung injury or infectious disease testing not performed (probable cases); and 4) absence of alternative plausible diagnoses. Most states are reporting case counts to CDC as case status is determined; however, it can take up to several weeks to complete and submit information from medical chart abstraction and interviews. Additional time might be required after the information is submitted to CDC to clean and standardize data submitted in different formats. This report summarizes patterns of the lung injury outbreak and characteristics of cases reported to CDC, including demographic characteristics and selected substances used by patients.^†^

As of September 24, 2019, 805 cases of lung injury from 46 states and one territory had been reported to CDC (Figure 1). Among the 805 cases reported, basic patient data (i.e., demographics and dates of symptom onset and hospitalization) were received for 771 (96%) patients. Ninety-one percent of patients were hospitalized. Median duration between symptom onset and hospitalization was 6 days (range = 0–158 days) (Figure 2). Although some cases occurred during April–June 2019, the number of cases began increasing in early July. The decline in reporting of onset dates and hospitalizations in the most recent 3–4 weeks is the result, in part, of a lag in reporting; there is no evidence that occurrence of lung injury cases is declining.

**FIGURE 1:**
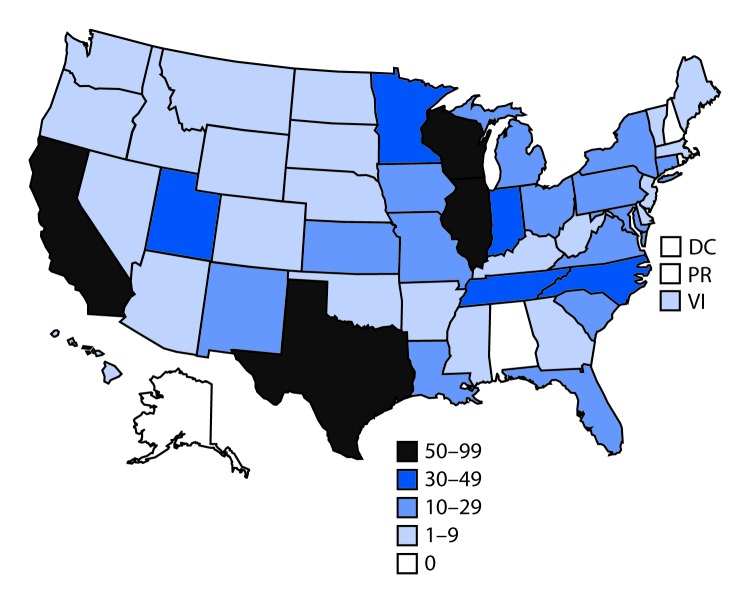
Number of cases of lung injury associated with e-cigarette use, or vaping (n = 805) — United States, including two territories, 2019* **Abbreviations:** DC = District of Columbia, PR = Puerto Rico; VI = U.S. Virgin Islands. *As of September 24, 2019, 1–9 cases had been reported by 23 states and one territory; 10–29 cases had been reported by 14 states; 30–49 cases had been reported by five states; 50–99 cases had been reported by four states, and 0 cases had been reported by four states and DC. Additional cases being investigated are not reflected on this map.

**FIGURE 2:**
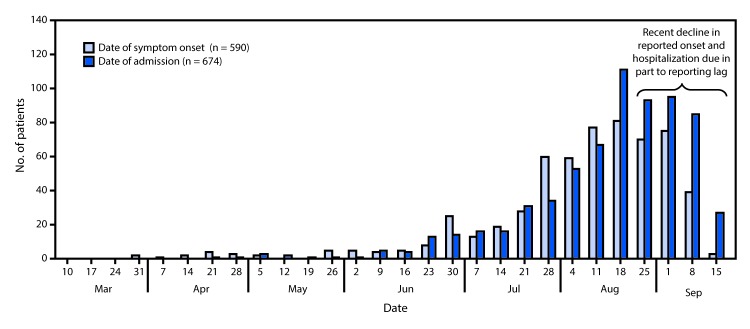
Dates of symptom onset (n = 590) and hospital admission (n = 674) among patients with lung injury associated with e-cigarette use, or vaping — United States, March 31–September 21, 2019

Sixty-nine percent of patients were male (Table). Median age was 23 years (range = 13–72 years); 61.9% were aged 18–34 years, and 16.2% were aged <18 years. Among the 12 deaths reported to CDC, 58% occurred in men, and the median age was 50 years (range = 27–71 years). Among a subset of 514 patients (63.8%) for whom information on substances used in e-cigarettes, or vaping, products was available, 395 (76.9%) reported using THC-containing products, and 292 (56.8%) reported using nicotine-containing products in the 30 days preceding symptom onset; 210 patients (40.9%) reported using both THC-containing and nicotine-containing products, 185 (36.0%) reported exclusive use of THC-containing products, and 82 (16.0%) reported exclusive use of nicotine-containing products. 

**TABLE:** Number of patients with lung injury associated with e-cigarette use, or vaping (n = 771), by demographic and substance use characteristics — United States, 2019

Characteristic	No. (%)
Demographic (n = 771)*	
**Sex**	
Male	531 (68.9)
Female	234 (30.4)
Missing	6 (0.8)
**Age group (yrs)**	
<18	125 (16.2)
18–24	293 (38.0)
25–34	184 (23.9)
35–44	93 (12.1)
≥45	42 (5.5)
Missing	34 (4.4)
**Substances used in e-cigarette, or vaping, products (n = 514)^†^**	
**THC-containing products**	
Yes	395 (76.9)
No	96 (18.7)
Unknown/Missing	23 (4.5)
**Nicotine-containing products**	
Yes	292 (56.8)
No	173(33.7)
Unknown/Missing	49 (9.5)
**Cannabidiol (CBD)**	
Yes	89 (17.3)
No	265 (51.6)
Unknown/Missing	160 (31.1)
**Synthetic cannabinoids**	
Yes	4 (0.8)
No	289 (56.2)
Unknown/Missing	221 (43.0)
**Flavored e-liquids** ^§^	
Yes	102 (19.8)
No	132 (25.7)
Unknown/Missing	280 (54.5)

**Abbreviation:** THC = tetrahydrocannabinol.

* Patients for whom basic demographic information was submitted to CDC.

^†^ Patients for whom information was available on use of either nicotine-containing or THC-containing substances.

^§^ Flavored products that contain water, food-grade flavoring, propylene glycol, vegetable glycerin, nicotine, THC, or CBD.

## Discussion

E-cigarettes were introduced to the U.S. market in 2007 (*1*). In 2018, 20.8% of high school students reported current e-cigarette use (*5*). E-cigarette use is markedly lower among U.S. adults than among youths; in 2018, only 3.2% of adults currently used e-cigarettes, with higher prevalences among persons aged 18–24 years (7.6%) and 25–34 years (5.4%) than among older age groups (*6*). Approximately three fourths of patients in this investigation were aged <35 years. In the general U.S. adult population, current e-cigarette use is slightly higher among males than females for both adults and youths (*6*); in the present investigation, approximately seven in 10 cases occurred in males. In this investigation, 62% of patients were aged 18–34 years; this is consistent with the age group with highest reported prevalence of marijuana use in the preceding 30 days in the United States (*7*).

THC-containing and nicotine-containing products were the most commonly reported substances used in e-cigarettes, or vaping products, by patients. Specific data on use of THC in e-cigarettes, or vaping products, in the general population is limited; among U.S. middle and high school students in 2016 who had ever used an e-cigarette, 30.6% reported using THC in an e-cigarette (33.3% among males and 27.2% among females) (*8*). Among adults who reported using marijuana in 2014, 9.9% reported consuming it via a vaporizer or other electronic device (11.5% among men and 7.8% among women) (*9*). In a recent study of college students, approximately 75% of those who had used substances other than nicotine in e-cigarettes reported using marijuana or THC-containing products in an e-cigarette (*10*). Because information about substance use in this investigation was self-reported, the information is not available for some cases because of the time required for completing and reporting patient interviews, inability to conduct interviews (e.g., patient refusal, loss to follow-up, persons who were too ill or died before they could be interviewed) and missing data for certain variables (e.g., patient refusal to answer certain questions). In addition, patients might not always know what substances they use or might be hesitant to reveal use of substances that are not legal in their state.

Continued monitoring of patient case counts and characteristics, as well as substances used with e-cigarette, or vaping, products, is critical to informing the ongoing investigation and helping to identify the cause. CDC and state health departments continue to collect and analyze epidemiologic data to better understand what types of devices and products patients are using (e.g., cartridges and e-liquids), the source of products or location where they were obtained, and the patterns (e.g., duration and frequency) of specific product use. Given the vast number of chemicals used in e-cigarette, or vaping, products, it is important to link epidemiologic data with findings from laboratory analyses of products and clinical specimens from patients. Federal, state, and private laboratories are working to collect and analyze products obtained from patients with lung injury associated with e-cigarette use, or vaping. In addition, CDC, clinical, and public health laboratories are collecting clinical specimens for future targeted analyses of substances identified in product samples.

The specific chemical exposure(s) causing this outbreak is unknown at this time. National data to date show that most lung injury patients with data on substance use report using THC-containing products with or without nicotine-containing products, although some patients report using only nicotine-containing products. While this investigation is ongoing, CDC recommends that persons consider refraining from using e-cigarette, or vaping, products, particularly those containing THC. Persons who continue to use e-cigarettes or vaping products should carefully monitor themselves and seek medical attention immediately if they have symptoms consistent with those described in this outbreak.^§^

Regardless of the investigation, e-cigarettes, or vaping products, should never be used by youths, young adults, pregnant women, or by adults who do not currently use tobacco products (*2*). Adults who use e-cigarettes because they have quit smoking should not return to smoking combustible cigarettes. In addition, persons who use e-cigarettes or vaping products should not get them from informal sources or off the street and should not modify e-cigarette, or vaping, devices or add any substances that are not intended by the manufacturer. Both THC-containing and nicotine-containing e-cigarette, or vaping, products purchased legally within states might also contain harmful substances (*1*); it is difficult for consumers to know what is in these products, and full ingredient lists are typically not available. THC use has been associated with a wide range of health effects, particularly with prolonged heavy use.^¶^ The best way to avoid potentially harmful effects is to not use THC, including through e-cigarette, or vaping, devices. Persons with marijuana use disorder should seek evidence-based treatment by a health care provider. 

This investigation is ongoing. CDC will continue to work in collaboration with FDA and state and local partners to investigate cases and advise and alert the public on the investigation as additional information becomes available.

**Summary**

**What is already known about this topic?**

Lung injury associated with e-cigarette use, or vaping, has recently been reported in most states. CDC, the Food and Drug Administration, and others are investigating this outbreak.

**What is added by this report?**

Among 805 cases reported as of September 24, 2019, 69% were in males; 62% of patients were aged 18–34 years. Among patients with data on substances used in e-cigarettes, or vaping products, tetrahydrocannabinol (THC)-containing product use was reported by 76.9% (36.0% reported exclusive THC-product use); 56.8% reported nicotine-containing product use (16.0% reported exclusive nicotine-product use).

**What are the implications for public health practice?**

The cause of the outbreak is unknown. While this investigation is ongoing, CDC recommends that persons consider refraining from using e-cigarette, or vaping, products, particularly those containing THC.

## Footnotes

* https://www.cdc.gov/tobacco/basic_information/e-cigarettes/assets/2019-Lung-Injury-Surveillance-Case-Definition-508.pdf.

† CDC determined the intent of this project to be public health practice for disease and injury control; thus, the activity is not research involving human subjects and Institutional Review Board approval was not required (OMB No. 0920–1011).

§ https://www.cdc.gov/tobacco/basic_information/e-cigarettes/severe-lung-disease/need-to-know/index.html.

¶ http://nationalacademies.org/hmd/reports/2017/health-effects-of-cannabis-and-cannabinoids.aspx.
