# *Erratum:* Vol. 68, No. 39

**DOI:** 10.15585/mmwr.mm6840a5

**Published:** 2019-10-11

In the report “Characteristics of a Multistate Outbreak of Lung Injury Associated with E-cigarette Use, or Vaping — United States, 2019,” on page 860, in the first paragraph, the ninth sentence should have read “Among 514 patients with information on substances used in e-cigarettes, or vaping products, in the **3 months** preceding symptom onset, 76.9% reported using THC-containing products, and 56.8% reported using nicotine-containing products; 36.0% reported exclusive use of THC-containing products, and 16.0% reported exclusive use of nicotine-containing products.”

Also on page 860, in the last paragraph, the third sentence should have read “Among a subset of 514 patients (63.8%) for whom information on substances used in e-cigarettes, or vaping, products was available, 395 (76.9%) reported using THC-containing products, and 292 (56.8%) reported using nicotine-containing products in the **3 months** preceding symptom onset; 210 patients (40.9%) reported using both THC-containing and nicotine-containing products, 185 (36.0%) reported exclusive use of THC-containing products, and 82 (16.0%) reported exclusive use of nicotine-containing products.”

